# Involvement of MicroRNA-296 in the Inhibitory Effect of Epigallocatechin Gallate against the Migratory Properties of Anoikis-Resistant Nasopharyngeal Carcinoma Cells

**DOI:** 10.3390/cancers12040973

**Published:** 2020-04-15

**Authors:** Chien-Hung Lin, Hsin-Hui Wang, Tsung-Hsien Chen, Ming-Chang Chiang, Peir-Haur Hung, Yann-Jang Chen

**Affiliations:** 1 Department of Pediatrics, Taipei Veterans General Hospital, Taipei 11217, Taiwan; chome0627@yahoo.com.tw (C.-H.L.); hhwang@vghtpe.gov.tw (H.-H.W.); yjchen@ym.edu.tw (Y.-J.C.); 2 Institute of Clinical Medicine, National Yang-Ming University, Taipei 11221, Taiwan; 3 Department of Life Science, College of Science and Engineering, Fu Jen Catholic University, New Taipei 242304, Taiwan; 089034@mail.fju.edu.tw; 4 Department of Pediatrics, Zhongxing Branch, Taipei City Hospital, Taipei 10341, Taiwan; 5 Institute of Emergency and Critical Care Medicine, National Yang-Ming University, Taipei 11221, Taiwan; 6 Department of Internal Medicine, Ditmanson Medical Foundation Chia-yi Christian Hospital, Chiayi 60002, Taiwan; cych13794@gmail.com; 7 Department of Applied Life Science and Health, Chia-Nan University of Pharmacy and Science, Tainan 71710, Taiwan; 8 Department of Life Sciences and Institute of Genome Sciences, National Yang-Ming University, Taipei 11221, Taiwan

**Keywords:** microRNA, epigallocatechin gallate (EGCG), nasopharyngeal carcinoma (NPC), cancer cell migration

## Abstract

Short noncoding endogenous RNAs, including microRNAs (miRNAs), are associated with the development and metastasis of multiple cancers. Epigallocatechin gallate (EGCG), the most active and abundant polyphenol in green tea, plays a crucial role in the modulation of miRNA expression, which is related to changes in cancer progression. In the present study, we explore whether EGCG exerts its suppressive effects on nasopharyngeal carcinoma (NPC) cells through miRNA regulation. The anoikis-resistant sphere-forming NPC cells grown under anchorage-independent conditions exhibit enhanced migratory properties, which were inhibited by EGCG treatment. The miR-296 level was lower in the anoikis-resistant cells than in the monolayer parental cells; however, miR-296 was significantly upregulated after EGCG treatment. We demonstrate that miR-296 is involved in the inhibitory effects of EGCG on the anoikis-resistant NPC cells through the downregulation of signal transducer and activator of transcription 3 (STAT3) activation. Our study is the first to demonstrate that EGCG inhibited the migratory properties of anoikis-resistant cells by modulating the expression of miRNA in NPC cells. Our results indicate the novel effects of EGCG on miRNA regulation to inhibit an invasive phenotype of NPC as well as the regulatory role of miR-296.

## 1. Introduction

Nasopharyngeal carcinoma (NPC) presents as tumors arising from the epithelial cells of the nasopharynx; it is highly prevalent in Southeast China and Taiwan. NPC is a complex disease in which genetic factors, environmental factors, and Epstein–Barr virus infection appear to play a major role [1]. NPC tumor cells have a high tendency to locally invade lymph nodes and metastasize to distant sites, causing poor outcomes in patients with NPC [2]. Additionally, these malignant NPC cells acquire anoikis-resistance (AR) to survive and proliferate after detaching from the original sites and then metastasize through the circulatory systems. Relapse and metastasis, which are common in NPC, hinder treatment. Therefore, it is necessary to explore effective therapeutic strategies to improve the prognosis of patients with NPC [3].

Epigallocatechin gallate (EGCG), a major polyphenol in green tea, has been shown to exhibit potential anticancer or cancer preventive effects [4,5]. Numerous studies have suggested that EGCG suppresses growth and induces apoptosis in certain cancer cells through various mechanisms. Furthermore, the inhibition of the epithelial–mesenchymal transition (EMT) by EGCG has been proposed to inhibit migration and metastasis in several types of tumors [6,7].

MicroRNAs (miRNAs) are small noncoding RNA molecules, which play major regulatory roles in normal biological processes, such as cell survival, proliferation, and apoptosis [8]. The aberrant expression of miRNAs may contribute to tumor initiation and is significantly associated with the evolution and progression of cancer. In tumorigenesis, miRNAs may function as either oncogenes or tumor suppressor genes [9]. Furthermore, recent studies suggested the involvement of miRNAs in the regulation of cancer stem cells and their association with EMT changes and the invasiveness of cancer cells [10,11,12].

Few reports have demonstrated that miRNAs are molecular targets for preventive and therapeutic strategies using several natural compounds [13,14]. Previous studies, including ours, reported that in NPC cells, EGCG inhibits growth as well as EMT, invasion, and migration [15,16,17]. However, it remains largely unknown whether miRNA expression is involved in the suppressive effects of EGCG on NPC cells.

In our study, we identified differential miRNA expression in NPC AR cells compared to the parental NPC cells. Certain miRNAs were significantly downregulated in NPC AR cells, and miR-296 was reversely upregulated by EGCG. The upregulation of miR-296 expression after EGCG treatment could lead to the suppression of the migratory properties of NPC, which are mediated by the inactivation of signal transducer and activator of transcription 3 (STAT3). Our results indicate a novel mechanism of action of polyphenolic compounds in green tea and suggest that EGCG may inhibit invasive metastatic activity of NPC through the regulation of miR-296 expression.

## 2. Results

### 2.1. EGCG Inhibits Enhanced Migratory Ability of Anoikis-Resistant NPC Cells

**Figure 1:**
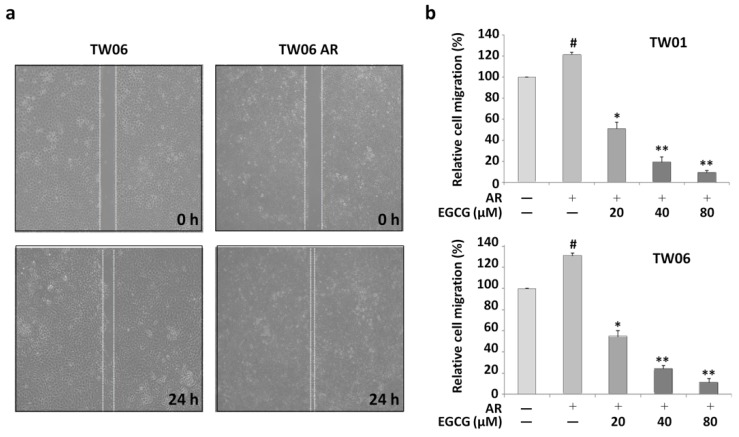
The enhanced migratory ability of the anoikis-resistant (AR) nasopharyngeal carcinoma (NPC) cells was suppressed by the effect of epigallocatechin gallate (EGCG). (**a**) A wound healing assay was performed for cell migration in parental cells and anoikis-resistant cells. (**b**) A transwell invasion assay was performed on the parental cells and anoikis-resistant cells following treatment with different concentrations of EGCG. * *p* < 0.01, ** *p* < 0.001 vs. anoikis-resistant control cells, and # *p* < 0.05 vs. parental cells. The data shown are represented as mean ± standard deviation (SD).

The migratory ability of AR cells was evidently higher than that of the parental adherent TW06 cell lines as shown in Figure 1a. However, after the cells were treated with different concentrations of EGCG (20, 40, and 80 μM) for 24 h, the transwell migration assay demonstrated that the enhanced migration of the AR cells was significantly inhibited (Figure 1b). These results indicate that although the NPC AR cells expressed aggressive behavior, their invasiveness was suppressed by the inhibitory effect of EGCG.

### 2.2. EGCG Induces miR-296 in Anoikis-Resistant NPC Cells

**Figure 2:**
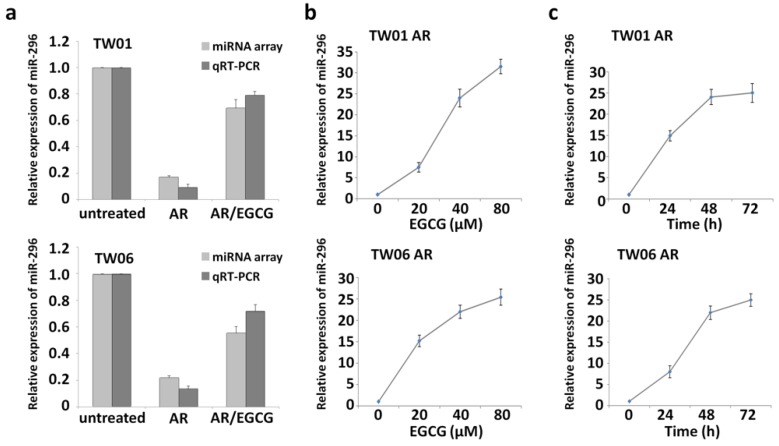
The miRNA expression analysis determined through miRNA array and quantitative real-time polymerase chain reaction (qRT-PCR). (**a**) The miRNA expression patterns of the nasopharyngeal carcinoma parental and anoikis-resistant (AR) cells with or without epigallocatechin gallate (EGCG) treatment were assessed using a miRNA array system. The miRNA array results were confirmed through qRT-PCR. (**b**) The qRT-PCR results revealed that miR-296 was significantly induced in the anoikis-resistant cells treated with EGCG for 48 h in a dose-dependent manner. (**c**) The qRT-PCR results revealed that miR-296 was significantly induced in the anoikis-resistant cells treated with 40 μM EGCG in a time-dependent manner.

To investigate the potential involvement of miRNAs in response to EGCG treatment, a miRNA array was performed to analyze the NPC AR cells treated with EGCG at 40 μM for 48 h as well as the corresponding untreated cells. In the comparison of the two differential miRNA profiles between EGCG-treated vs. untreated NPC AR cells and NPC AR cells vs. the parental cells, the results show that the expression of miR-296 and miR-328 was expressed at higher levels (ΔΔCt value decrease) after the effect of EGCG in the NPC AR cells, which was originally downregulated compared to the parental cells (Figure S1). This finding implicates that these two miRNAs may play a particular role in the effects of EGCG on NPC invasiveness. The data revealed that the miR-296 levels were significantly lower in both the TW01 and TW06 AR cells than in the parental cells; however, EGCG treatment upregulated miR-296 (Figure 2a). The expression levels of miR-296 were confirmed through real-time PCR and consistent with those in miRNA array analysis (Figure 2a). Furthermore, miR-296 was induced by EGCG in both a dose- and time-dependent manner (Figure 2b,c). These data demonstrate that although miR-296 expression decreased in the NPC AR cells under nonadherent growth, miR-296 levels could be elevated in response to the effect of EGCG.

### 2.3. Overexpression of miR-296 Inhibits Cell Migration of Anoikis-Resistant NPC Cells

**Figure 3:**
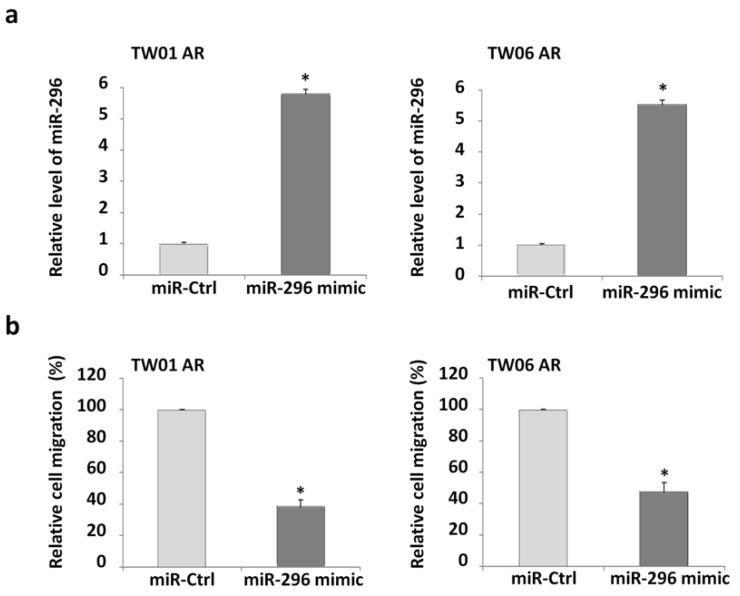
Overexpression of miR-296 affects the migratory ability of the anoikis-resistant nasopharyngeal carcinoma cells. (**a**) Anoikis-resistant (AR) cells were transfected with a miR-296 mimic, and the expression levels of miR-296 were analyzed through qRT-PCR. (**b**) The transwell assay was used to evaluate cell migratory ability. The data show the relative cell counts calculated and normalized to those of the control treatment, measured in triplicate and presented as mean ± SD (* *p* < 0.05).

To determine whether the upregulation of miR-296 induced by EGCG exerted inhibitory effects on the cell migration of NPC AR cells, we supplemented the cells with an exogenous source of miR-296 by transfecting an miR-296 mimic in the TW01 and TW06 cells (Figure 3a). As expected, transfection of the miR-296 mimic significantly suppressed the migration of the NPC AR cells (Figure 3b). These results suggest that the increase in miR-296 expression caused by EGCG inhibited the invasiveness of the NPC AR cells.

### 2.4. miR-296 is Involved in the Inhibitory Effects of EGCG through Downregulation of STAT3 Expression

**Figure 4:**
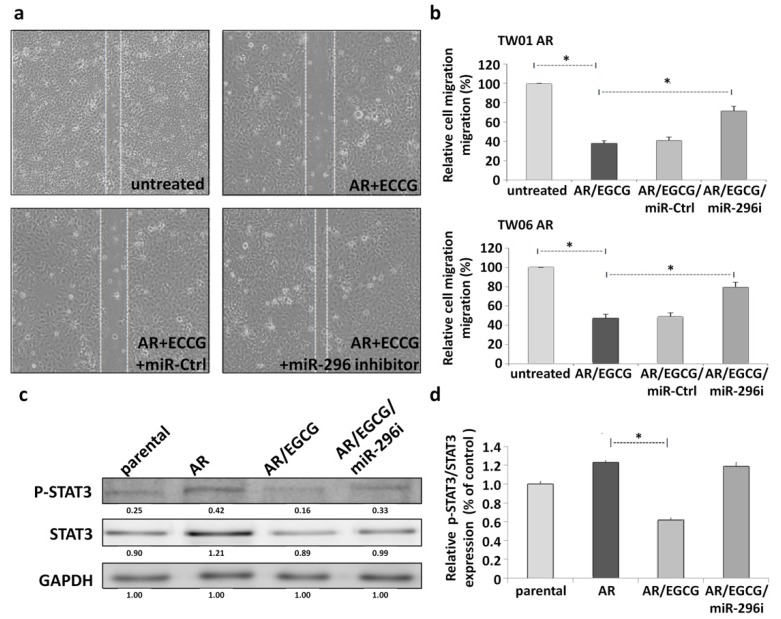
Knockdown of miR-296 attenuates the inhibitory effect of epigallocatechin gallate (EGCG) on the anoikis-resistant nasopharyngeal carcinoma cells. (**a**) The wound-healing method demonstrated the migratory behaviors of the TW06 anoikis-resistant (AR) cells after the effect of EGCG with or without transfection of the miR-296 inhibitor. (**b**) The transwell assay demonstrated the migration of the anoikis-resistant cells after the EGCG treatment with or without transfection of the miR-296 inhibitor (miR-296i) (* *p* < 0.05). (**c**) Western blot analysis of the signal transducer and activator of transcription 3 (STAT3) and phosphorylated STAT3 (p-STAT3) expression of the TW06 anoikis-resistant cells after treatment with EGCG with or without transfection of the miR-296 inhibitor (miR-296i). (**d**) The relative protein levels were quantified by densitometry, and the p-STAT3/total STAT3 ratio using normalized values was expressed as the relative density ratio with control glyceraldehyde 3-phosphate dehydrogenase (GAPDH).

To determine whether EGCG suppresses the migration of NPC AR cells through upregulation of miR-296, the TW01 and TW06 AR cells were transfected with an miR-296 inhibitor and subsequently treated with EGCG. The results of the wound-healing (Figure 4a) and transwell migration (Figure 4b) assays show that transfection with the miR-296 inhibitor significantly reduced the EGCG-induced inhibition of migration of the NPC AR cells. This finding reveals that miR-296 may serve as a novel target of EGCG and is involved in the EGCG-inhibited cell migration of the NPC AR cells.

STAT3 is evidently activated in the NPC AR cells and plays a critical role in the development of enhanced migratory ability [18]. To determine whether EGCG inhibits the migration of NPC through the regulation of miR-296 and STAT3, we analyzed the STAT3 expression in the NPC AR cells that were transfected with the miR-296 inhibitor and subsequently treated with EGCG. The results of Western blot analysis of STAT3 and phosphorylated STAT3 (p-STAT3) accumulation show that the total STAT3 protein expression was highly enhanced in the NPC AR cells; in the EGCG-treated NPC AR cells, it decreased back to the parental level (both total and phospho); whereas in the EGCG-treated NPC AR cells plus the miR-296 inhibitor, this decrease was lower (Figure 4c,d). The EGCG-inhibited STAT3 expressions of the NPC AR cells were significantly attenuated after transfection with the miR-296 inhibitor. This result implied that miR-296 was involved in the inhibitory effect of EGCG on p-STAT3 modification, which was activated in NPCs with an invasive phenotype.

## 3. Discussion

NPC is characterized by high rates of regional lymph node invasion and systemic metastases. Anoikis-resistant cancer cells possess an invasive phenotype and exhibit gene expression patterns that are distinct from those of the parental cells [19,20]. Some NPC cells that develop resistance to anoikis can exhibit sphere formation and acquire enhanced migration and invasion abilities.

Accumulating evidence shows that natural agents, such as dietary polyphenols (curcumin, resveratrol, and tea polyphenols), have chemopreventive effects and may be combined with conventional therapy for more effective treatment of cancer [21,22,23]. Green tea polyphenols, such as EGCG, exhibit potential activity against various tumor cells through the regulation of multiple signaling pathways. Previous reports demonstrated that EGCG could regulate protein kinases and phosphatase to induce apoptosis and inhibit the tumorigenesis of cancer cells [24,25,26]. Some reports suggest that certain candidate miRNAs may be potential molecular targets of EGCG, which is appreciated as a possible mechanism for its anticancer activity [27,28]. In the present study, we found that EGCG inhibited the invasive characteristics of the NPC AR cells, and the inhibitory effect was associated with alterations in the expression of miRNAs.

Comparing the expression of miRNAs in the NPC cells grown in two culture conditions confirmed that the miR-296 levels were significantly lower in the NPC sphere cells growing nonadherently compared with the monolayer parental cells. The downregulation of miR-296 was associated with anoikis resistance and may promote NPC cell survival and metastasis. This was supported by the observation that the NPC cells transfected with an miR-296 mimic resulted in reduced migration. These findings may indicate that the status of miR-296 expression may be closely associated with the invasiveness of NPC cells.

Moreover, we demonstrated that the inhibitory effects of EGCG on the migratory properties of NPC cells were associated with the upregulation of miR-296, which was downregulated in the NPC AR cells. Furthermore, the miR-296 upregulation by EGCG occurred in a dose- and time-dependent manner in the TW01 and TW06 cells. This result implied that EGCG may induce miR-296 to inhibit the migration of the NPC AR cells. Additionally, we found that EGCG-induced inhibition of cell migration of the NPC cells was significantly attenuated after transfection with the miR-296 inhibitor. These results indicated the role of miR-296 in regulating the migratory properties of the NPC cells and also that miR-296 expression in the NPC cells may be modulated by EGCG for suppression of the aggressive behavior of NPC cells.

Dysregulated expression of miR-296 has been associated with several human cancers [29,30]. Studies demonstrated that upregulation of miR-296 levels was associated with tumor growth and correlated with advanced stages in several types of cancers [31,32]. By contrast, miR-296 may also play a crucial role in tumor suppression and could regulate its target genes to inhibit the invasive properties of cancer cells, including migration and invasion. Consistent with our results, underexpression of miR-296 may cause tumor progression by inducing EMT events in some tumor cells [33,34]. miR-296 negatively regulated by nicotine directly targets mitogen-activated protein kinase-activated protein kinase-2-induced Ras/Braf/Erk/Mek/c-Myc or Phosphoinositide 3-kinase (PI3K)/Protein Kinase B(AKT)/c-Myc signaling to stimulate its own expression and suppress NPC cell proliferation and metastasis [35].

The critical involvement of STAT3 in survival and proliferation of tumor cells has been previously reported [36,37]. STAT3 is an important molecule in the Janus kinase signal transducer pathway and the activator of transcription signal pathways, and plays critical roles in cell proliferation, metastasis, angiogenesis, host immune evasion, and therapy resistance. STAT3 normally resides in the cytosol, but the activated STAT3 (p-STAT3) complex translocates into the nucleus to initiate the transcription of STAT3 target genes, including the genes encoding cyclin D1, Bcl-xL, c-Myc, Mcl1, survivin, and vascular endothelial growth factor [38,39,40,41,42].

Increased STAT3 activation (p-STAT3) was found to play a role in driving NPC progression and metastasis and was found to be clinically associated with advanced stages (stage III or IV) of NPC [43]. The detrimental effect of EGCG on the phosphorylation of STAT3 has been described in hepatocellular carcinoma cell lines and colorectal carcinoma [44,45]. Furthermore, an increasing number of studies suggest that STAT3 plays a crucial role in the regulation of anoikis resistance and invasion of cancer cells [46,47,48]. Based on our previous findings, we investigated whether the NPC AR cells were more invasive than parental cells and whether the NPC AR cells exhibited significantly higher expression of STAT3 with enhanced downstream gene expression than the parental cells [18].

In this study, we investigated the EGCG inhibition of the invasive characteristics of NPC AR cells, through regulating the expression of miR296 and STAT3 activation. These findings indicated that miR-296 is a crucial modulator of one of the intrinsic pathways in EGCG governing the anoikis resistance of NPC, resulting in a STAT3 blockade, and suppressing NPC cell migration and invasion. Our study identified that EGCG could interfere with STAT3 signaling activation in NPC cells through a novel signaling axis of miR-296/STAT3 in enhancing anoikis resistance, suggesting that miR-296/STAT3 may serve as a potential therapeutic target for metastatic NPC.

## 4. Methods

### 4.1. Cell Culture

Parental cell culture: Two human NPC cell lines, TW01 (keratinizing squamous cell carcinoma) and TW06 (undifferentiated carcinoma), were cultured in 10 cm^2^ dishes with Dulbecco’s modified Eagle’s medium (DMEM; GIBCO/Invitrogen, Carlsbad, CA, USA) and 10% fetal bovine serum, 1% sodium pyruvate, 1% penicillin/streptomycin/amphotericin, and 1% nonessential amino acids, as described previously [49]. The cells were incubated at 37 °C in a humidified atmosphere comprised of 95% air and 5% CO_2_.

Nonadherent culture: The NPC TW01 and TW06 cells were seeded nonadhesively in 6-well culture dishes coated with a thin layer of agarose at a density of 2 × 10^4^/mm^3^ in serum-free DMEM/F12 medium (GIBCO). The NPC AR cells were maintained for 7 to 10 days with complete media changes every other day during this period, as described previously [50]. These anoikis-resistant sphere-forming NPC cells were collected using filtration through a 70-μm mesh for subsequent experiments.

### 4.2. Cell Migration Analysis

The analysis of cell migration was performed using wound-healing and transwell migration assays. The wound-healing assay was performed in 6-well plates using the cells incubated previously under either adherent or nonadherent conditions. The cells were grown to confluence and were photographed after scratching with a sterile 10 µL tip at 0 and 24 h under the necessary conditions. Transwell migration assays were performed in BD Falcon cell culture inserts (BD Biosciences, San Jose, CA, USA); 10^5^ cells suspended in 100 mL of serum-free DMEM were seeded in the upper compartment, and the lower compartments were filled with DMEM containing 10% fetal bovine serum (FBS). After 24 h of incubation, the migrated cells on the reverse side were fixed with methanol and stained with 0.1% crystal violet. The migrated cells were counted under a light microscope in five randomly selected fields (magnification, ×100). Once the total number of cells per insert was determined, the percent migration/invasion was calculated by dividing this number by the number of cells seeded. All the samples were normalized to the parental cells’ migration (parental = 100%).

### 4.3. MiRNA Array Analysis

For the miRNA cDNA synthesis, the RNA was reverse transcribed using the Megaplex RT Primer, Human Pool Sets and was preamplified using the Megaplex PreAmp Primer, Human Pool Sets (Applied Biosystems, Waltham, MA, USA). The resulting cDNAs were loaded onto TaqMan Low-Density miRNA Array (TLDA) cards according to the manufacturer’s instructions for the study of miRNA expression and these were run on an ABI 7900HT Real-Time PCR system. The TLDA analysis expression values were calculated using the comparative threshold cycle (CT) method. This technique uses the formula 2^−ΔΔCT^ to calculate the expression of the target miRNAs normalized to a calibrator. The CT indicates the cycle number at which the amount of amplified target reaches a fixed threshold. The CT values ranged from 0 to 36. The ΔCT values (ΔCT = CT (target miRNA) − CT (U6)) were calculated for the control sample and were subsequently used as the calibrator, for which all miRNA expression values were assigned a relative value of 1.00, to determine the comparative miRNA expression so that ΔΔCT = ΔCT (test sample) − ΔCT (control sample).

### 4.4. Small RNA Extraction and Quantification of miR-296 through Quantitative Real-Time Polymerase Chain Reaction (qRT-PCR)

The total RNA was extracted using the mirVana miRNA Isolation Kit (Applied Biosystems) following the manufacturer’s instructions and quantified using NanoDrop (Thermo Fisher Scientific Inc, Waltham, MA, USA). The miRNAs were reverse-transcribed using the TaqMan microRNA Reverse Transcription Kit (Applied Biosystems). The primer for miR-296 was designed according to the miRBase Sequence Database (ID: hsa-miR-296-3p, 5′-GAGGGUUGGGUGGAGGCUCUCC-3′), and synthesized by Invitrogen. The sequence-specific forward primer for the U6 internal control was 5′-CTCGCTTCGGCAGCACATATA-3′. Quantification was performed by real-time PCR using the ABI PRISMW 7900HT system (Applied Biosystems, Foster City, CA, USA). The RT-qPCR protocol was performed according to the Single TaqMan microRNA assay (Applied Biosystems) instructions. The PCR cycling program consisted of 35 cycles of 30 s at 94 °C, 55 °C for 30 s, and 72 °C for 45 s. All PCR reactions were done in triplicate and all experiments were repeated at least three times. The default threshold settings were used as CT data. The CT is the fractional cycle number at which the fluorescence passes the fixed threshold.

### 4.5. miRNA Mimic or Inhibitor Transfection

Transfection was performed using the GenMute siRNA Transfection Reagent (SignaGen Laboratories, Rockville, MD, US) according to the manufacturer’s instructions. The negative control as well as miR-296 mimic and inhibitor were purchased from Dharmacon (Lafayette, CO, USA). The transfection buffer was diluted, incubated to a final concentration of 10 nM of miR-296 mimic or inhibitor for 15 m at room temperature, and then added to each well. We seeded 2 × 10^4^ cells on 6-well plates and incubated them at 37 °C with 5% CO_2_ for 24 h. The cells were incubated in fresh culture medium 30 min before the transfection. After 24 h, the cells were transfected with a miR-296 mimic or inhibitor and the negative control according to the manufacturer’s protocol.

### 4.6. Western Blot Analysis

Western blot analysis was performed as described previously [15]. The cells were lysed and sonicated in the radioimmunoprecipitation (RIPA) lysis buffer containing protease inhibitor. The protein concentrations were determined using a bicinchoninic acid (BCA; Thermo Fisher Scientific Inc) Protein Assay Kit. Equal amounts of proteins were separated through 10% sodium dodecyl sulfate–polyacrylamide gel electrophoresis (SDS-PAGE) by electrophoresis, and then samples were transferred onto a polyvinylidene fluoride (PVDF) membrane. After blocking the PVDF membrane with 5% nonfat dried milk, followed by immunoblotting with the STAT3 (Sigma-Aldrich S5933, St. Louis, MO, USA), phosphorylated STAT3 (Tyrosine 705, Sigma-Aldrich S4933), and glyceraldehyde 3-phosphate dehydrogenase (GAPDH, Sigma-Aldrich G9545) antibodies, respectively, we then incubated the membrane with secondary antibodies conjugated with horseradish peroxidase. The immune complexes on the PVDF membrane were detected using an Electrochemiluminescence (ECL) detection system. Uncropped blots are provided in Figure S2.

### 4.7. Statistical Analysis

The data represent the mean ± standard deviation (SD) of a minimum of three independent experiments, each performed in triplicate, and are presented relative to the control. Images were analyzed by Image J software, using the subtraction background method. Comparisons between the two groups were made using Student’s *t*-test, and *p*-values less than 0.05 were considered statistically significant.

## 5. Conclusions

Upregulated miR-296 expression induced by EGCG inhibited the invasiveness of NPC through inactivation of STAT3, which exhibited high expression in the NPC AR cells. We concluded that EGCG-induced suppression of the migratory properties of NPC cells was associated with the modulation of miRNA expression, primarily of the key regulator, miR-296, which may mediate the EGCG inhibition effect.

## Supplementary Materials

The following are available online at https://www.mdpi.com/2072-6694/12/4/973/s1, Figure S1: The miRNA levels were assayed by TaqMan Low-Density Array (TLDA) using the ΔΔCT method to calculate the expression, Figure S2: Uncropped immunoblots.
