# Supplementary material for: Involvement of MicroRNA-296 in the Inhibitory Effect of Epigallocatechin Gallate against the Migratory Properties of Anoikis-Resistant Nasopharyngeal Carcinoma Cells
Source: Cancers (Basel). 2020 Apr 15;12(4):973. doi: 10.3390/cancers12040973 (PMC7226234; doi:10.3390/cancers12040973)
Supplement: Supplementary file 1 [file cancers-12-00973-s001.pdf]

Supplementary Materials

Involvement of MicroRNA-296 in the Inhibitory Effect of Epigallocatechin Gallate against the Migratory Properties of Anoikis-Resistant Nasopharyngeal Carcinoma Cells

Chien-Hung Lin, Hsin-Hui Wang, Tsung-Hsien Chen, Ming-Chang Chiang, Peir-Haur Hung and Yann-Jang Chen

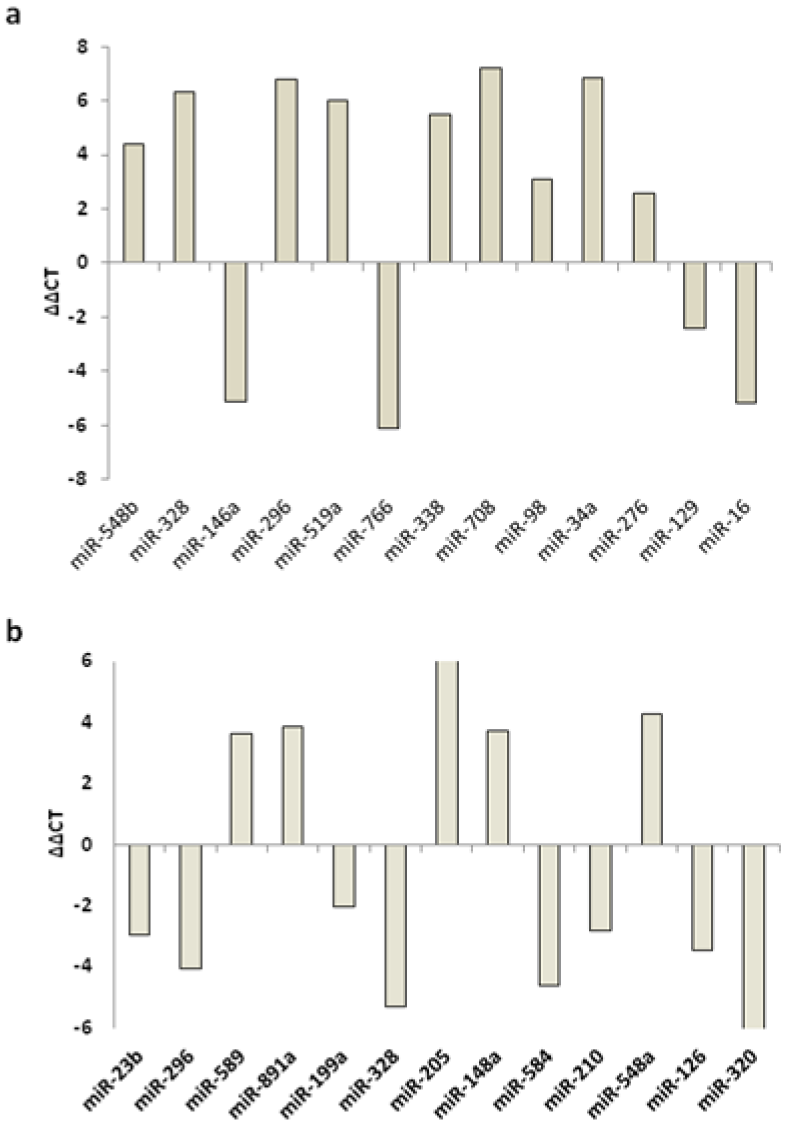

Figure S1. The miRNA levels were assayed by TaqMan Low-Density Array (TLDA) using the ΔΔCT method to calculate the expression. (**a**) The bar of each miRNA represents the results of the NPC AR cells comparing to the parental cells (Onco. Targets Ther. 2018; 11: 2375–2385, Figure S1) (**b**) The bar of each miRNA represents the results of the NPC AR cells treated with EGCG and the corresponding untreated NPC AR cells normalized to endogenous controls. The data showed that the miR-296 and miR-328 were reversely expressed after EGCG treatment in NPC AR cells.

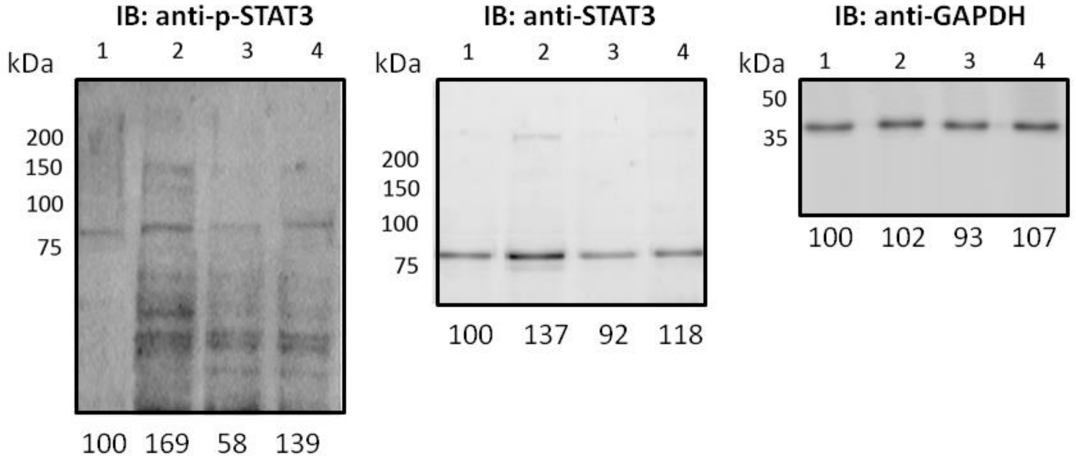

Figure S2. Uncropped immunoblots. In Figure 4c, PVDF membranes were cut following protein transfer, based on protein markers, to facilitate comparative analysis of protein levels by parallel probing of primary antibodies. The numbers below the graph indicate densitometry readings of full lanes relative to respective parental loading control. Line 1, parental; Line 2, AR; Line 3, AR/EGCG; Line 4, AR/EGCG/miR-296i.

© 2020 by the authors. Licensee MDPI, Basel, Switzerland. This article is an open access article distributed under the terms and conditions of the Creative Commons Attribution (CC BY) license (http://creativecommons.org/licenses/by/4.0/).
